# Type I Interferon Induced and Antagonized by Foot-and-Mouth Disease Virus

**DOI:** 10.3389/fmicb.2018.01862

**Published:** 2018-08-13

**Authors:** Xiao-xia Ma, Li-na Ma, Qiu-yan Chang, Peng Ma, Lin-Jie Li, Yue-ying Wang, Zhong-ren Ma, Xin Cao

**Affiliations:** ^1^Center for Biomedical Research, Northwest Minzu University, Lanzhou, China; ^2^State Key Laboratory of Veterinary Etiological Biology, Lanzhou Veterinary Research Institute, Chinese Academy of Agricultural Sciences, Lanzhou, China

**Keywords:** type I interferon, *Picornaviridae*, FMDV, RIG-I, MDA5

## Abstract

Viral infections trigger the innate immune system, serving as the first line of defense, and are characterized by the production of type I interferon (IFN). Type I IFN is expressed in a broad spectrum of cells and tissues in the host and includes various subtypes (IFN-α, IFN-β, IFN-δ, IFN-ε, IFN-κ, IFN-τ, IFN-ω, IFN-ν, and IFN-ζ). Since the discovery of type I IFN, our knowledge of the biology of type I IFN has accumulated immensely, and we now have a substantial amount of information on the molecular mechanisms of the response and induction of type I IFN, as well as the strategies utilized by viruses to evade the type I IFN response. Foot-and-mouth disease virus (FMDV) can selectively alter cellular pathways to promote viral replication and evade antiviral immune activation of type I IFN. RNA molecules generated by FMDV are sensed by the cellular receptor for pathogen-associated molecular patterns (PAMPs). FMDV preferentially activates different sensor molecules and various signal transduction pathways. Based on knowledge of the virus or RNA pathogen specificity as well as the function-structure relationship of RNA sensing, it is necessary to summarize numerous signaling adaptors that are reported to participate in the regulation of IFN gene activation.

## Introduction

Foot-and-mouth disease virus (FMDV) belongs to the *Aphthovirus* genus in the *Picornaviridae* family, and a highly infection disease caused by FMDV is regarded as an important concern for animal health (Knight-Jones et al., 2016). During FMDV evolutionary process, high mutation rates of the viral genome and quasispecies dynamics are considered major genetic factors (Shih et al., 1992). Thus, a series of studies were conducted to examine the relationship between genetic changes of the viral genome and viral fitness and different host/viral pathogenicities. Except for positive/negative selection and the random drift of the genome (Domingo et al., 2003), synonymous codon usage patterns of the FMDV genome also dominate its host ranges and viral proteins with normal biological functions (Zhou et al., 2010a,b, 2011, 2013a,b,c; Ahn et al., 2011; Ma et al., 2013; Ma X.X. et al., 2016; Gao et al., 2014). Due to the high genetic diversity of FMDV, the measures for controlling this disease need to be developed comprehensively, including killing infected and in-contact animals, the limitation of animal movement and vaccination based on conventional vaccines or new typical ones (Robinson et al., 2016). To further improve measures involved in antiviral treatments and novel vaccines for controlling rapid FMDV spread, it is important to obtain a deep understanding of the interaction between the host and FMDV. The antiviral immune response is the major focus on resisting FMDV infection, including innate/adaptive immune activations (Golde et al., 2008; Toka and Golde, 2013). The innate immune system serves as the first line of defense for resisting viral infections. The rapid induction of type I interferon (IFN) and other antiviral cytokines at the site of infection are part of the defense involved in antiviral immunity. The type I IFN family of placental mammals comprises 9 recognized classes identified to date: IFN-α, IFN-β, IFN-δ, IFN-ε, IFN-κ, IFN-τ, IFN-ω, IFN-ν, and IFN-ζ (Krause and Pestka, 2005; Detournay et al., 2013).

Type I IFNs exhibit direct antiviral activities by inhibiting viral replication and mediating the cellular immune functions of both the innate and adaptive immune system, resulting in both early limitation of the virus and long-term immunity. However, viruses are capable of selecting various strategies to evade the host immune system and thus contributing to viral pathogenicity (Schulz and Mossman, 2004; Jackson et al., 2017; Sumner et al., 2017). For FMDV infection, type I IFNs also play important roles in counteracting viral infection represent a potent biotherapeutic method against FMDV (Rodríguezpulido et al., 2011; Borrego et al., 2017). This minireview summarizes the current knowledge on how type I IFN is resistant to FMDV infection and how FMDV counteracts type I IFN induction and signaling transduction to evade the type I IFN system of host.

## Recognition of Viral Genome for IFN Production

**FIGURE 1:**
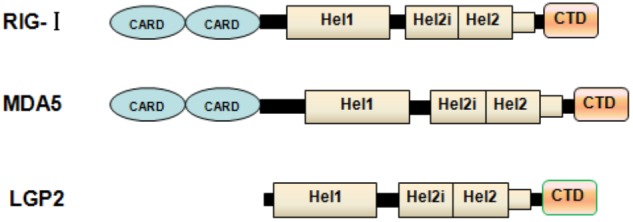
Physical organization of RLs. Schematic representation of RLR domains. The caspase activation and recruitment domain (CARD), helicase domain and RNA recognition domain are shown. LGP2 lacks the N-terminal CARD domain used by MDA5 and RIG-I for signal transduction.

Once viral infection occurs, cells of the infected host can trigger a series of activations of cytokines, including type I and type III IFNs. These IFNs can perform multiple biological functions related to antiviral, antiproliferative and immunomodulatory activations and trigger various interferon stimulated genes (ISGs), thereby contributing to the establishment of the antiviral state in which various steps of viral replication are restricted (Sen and Sarkar, 2007; Fensterl et al., 2015). Notably, both type I and type III IFNs represent similar patterns of expression and mechanisms of induction (Uzé and Monneron, 2007). A pivotal feature of IFN expression is the requirement for detection of the invading pathogens by pathogen-associated receptors. Generally, the innate immune system relies on germ-line-encoded pattern recognition receptors (PRRs) to recognize non-self RNA (viral RNA) which is one of the pathogen-associated molecular patterns (PAMPs) (Akira et al., 2006). For recognizing the viral RNA genome by the PRRs, there are two major classes of PRRs: Toll-like receptor (TLRs) at the cell surface or in endosomes, and retinoic acid-inducible gene-I (RIG-I) like receptors (RLRs) (Akira et al., 2006; Bruns et al., 2014). Among TLRs related to antiviral immune response, TLR 3 can recognize double-stranded RNA (ds RNA) and TLRs 7/8 can recognize single-stranded RNA (Alexopoulou et al., 2001; Diebold et al., 2004; Heil et al., 2004). However, TLR3 can sense ssRNA of poliovirus in some conditions (Tatematsu et al., 2013). Turning to RIG-I like receptor, which is involved in the antiviral immune response (**Figure 1**), melanoma differentiation-associated gene 5 (MDA5), laboratory of genetics and physiology 2 (LGP2) and RIG-I, which are ubiquitous cytosolic RNA helicases, play a pivotal role in recognizing viral RNA fragments (Yoneyama et al., 2005). It has been accepted that RIG-I can preferentially sense short dsRNAs, while MDA5 can recognize long dsRNA (Kato et al., 2008; Berke et al., 2013; Liu et al., 2016). Compared with the physical organizations and biological functions of MDA5 and RIG-I, LGP2 loses its CARD domain and displays a regulatory role that works as a concentration dependent biphasic status to mediate activations of MDA5 (Bruns et al., 2013, 2014; Uchikawa et al., 2016). Interestingly, RIG-I deficiency does not inhibit the replication of *Picornaviridae*, while MDA5 displays a remarkable role in resisting these viruses (Kato et al., 2006). Depending on the mice model with LGP2 deletion, dendritic cells (DCs) derived from these mice fail to generate IFN production upon infection by encephalomyocarditis virus (EMCV), vesicular stomatitis virus (VSV), and Newcastle disease virus (NDV) (Venkataraman et al., 2007; Satoh et al., 2010), suggesting that LGP2 might play a positive role *in vitro* by recognize some viral infections to promote IFN production.

## Recognition of FMDV RNA by TLRS and RLRS

The differential viral recognition by RIG-I and MDA5 is partly due to the differential recognition of distinct types of RNA patterns. It has been reported that MDA5 can recognize ssRNA involved in EMCV, Theiler’s virus and Mengo virus to activate IFN systems (Kato et al., 2006). Like those (+) ssRNA viruses mentioned above, FMDV RNA was recognized by MDA5 rather than by TLR3 or RIG-I in porcine epithelial cells, based on the result of the porcine kidney (PK-15) cells with MDA5 knockdown by RNA interference (Hüsser et al., 2011). According to the current knowledge on the length of RNA sensed by RIG-I and MDA5 (Kato et al., 2008), the FMDV genomes longer than 8000 nt are proposed to be sensed by MDA5 but not by RIG-I. Even though FMDV breaks into target cells and inhibits host translational systems by generation of L^pro^ and 3C^pro^ to evade the innate immune response against viral infection, MDA5 can first initiate IFN-β expression regardless of impairment of host translational systems. In addition, EMCV can produce high molecular weight RNA with a single- and double-stranded structure, and this RNA structure is capable of triggering MDA5 (Pichlmair et al., 2009). Notably, similar RNA structure exists in the 5′untranslation region of the FMDV genome (Carrillo et al., 2005), hence contributing to the activation of MDA5 to some degree. Most recently, it has been reported that the S fragment in the 5′untranslation region (UTR) of the FMDV genome is required for the replication and modulation of the innate immune response in host cells (Kloc et al., 2017). In human HEK 293 cell line, the S fragment and internal ribosomal entry site of 5′UTR and 3′UTR in the FMDV genome can trigger IFN-β promoter activation (Borrego et al., 2015). IFN-α/β can trigger double-stranded RNA-dependent protein kinase R (PKR) in the direct inhibition of FMDV replication *in vitro* (Chinsangaram et al., 2001; De et al., 2006). Turning to FMDV infection recognized by TLRs, the expression of TLR-4 in nasal-associated lymphoid tissue (NALT) during the acute stage of FMDV infection is higher than that of uninfected cattle, but non-infected and infected cattle do not differ regarding the transcription levels between TLR3 and TLR4 in NALT (Zhang et al., 2006). In addition, the FMDV genome can be recognized by TLR7 and TLR9 in plasmacytoid dendritic cells (pDCs) (Guzylack-Piriou et al., 2006; Lannes et al., 2012).

## FMDV-Induced Signaling Cascade and IFN Gene Activation

After RIG-I or MDA5 bind to viral RNA, the tandem CARDs are unmasked and interact with MAVS (also termed as Cardif, IPS-1 or VISA), which contains one CARD, by CARD-CARD interaction (Kawai et al., 2005; Meylan et al., 2005; Seth et al., 2005; Xu et al., 2005). MAVS are located on the outer membrane of the mitochondria and this specific localization is pivotal for signal transduction, because the formation of the RLR-MAVS complex contributes to the recruitment of numerous signaling adaptors (Seth et al., 2005). Recently, Sun Hur and her colleagues have postulated a novel mechanism showing that two CARD tetramer formation is essential for triggering the MAVS prion-like structure, leading to type I interferon production (Wu and Hur, 2015). Notably, the Atg5-Atg12 conjugate, an essential regulator of the autophagic machinery, directly associates with the CARD of the RIG-I and IFN-β promoter (also called IPS-1 or MAVS) and further impair the interaction between RIG-I and MAVS, hence contributing to RNA virus replication in host cells (Jounai et al., 2007). Interestingly, FMDV 3C^pro^ can degrade the Atg5-Atg12 complex to suppress autophagy and antiviral responses mediated by the NF-κB pathway (Fan et al., 2017). However, it needs to be noted that FMDV L^pro^ can degrade NF-κB as well and decrease IRF3/7 expression to suppress dsRNA-induced type I IFN production, thereby impairing the expression of IFNs (Wang et al., 2010, 2012). Taken together, FMDV can adopt multiple measures to go against the antiviral immune response. IFNs are induced through transcriptional mechanisms involving the transcription factors nuclear factor (NF)-κB and the IFN regulatory factors (IRFs). The RLR-MAVS complex can recruit TRAF (tumor necrosis factor receptor-associated factor) family members and transmit signals to downstream protein kinases (inhibitor of NF-κB (IκB) kinase (IKK) family members, which are pivotal for activating the transcription factors IRF-3, IRF-7, and NF-κB (Karin and Benneriah, 2000; Xu et al., 2005), thereby contributing to IFNs expression. As an ovarian tumor domain (OUT)-containing enzyme, de-ubiquitinating enzyme (DUB) A interacts directly with TRAF3 and catalyzes the cleavage of Lys-63-linked ubiquitin chains of TRAF3, thereby contributing to the dissociation of TBK1 from TRAF3 and blocking signal transductions mediated by RLRs (Kayagaki et al., 2007). During FMDV evolution, viral L^pro^ evolved a specific function domain which is similar to the cellular DUBs and possesses DUB activity to impair signal transduction related to type I IFN production (Wang et al., 2011b). The canonical IKK complex, which is composed of IKK-α, IKK-β and the regulatory subunit NF-κB essential modulator (NEMO, or called IKK-γ), phosphorylates IκB, and subsequent proteasome-dependent degradation of IκB results in functional NF-κB moving into the nucleus (Karin and Benneriah, 2000). In contrast, non-canonical IKKs, TRAF family associated NF-κB activator (TANK)-binding kinase 1 (TBK1), and IKK-iii, activate the signal-dependent phosphorylation of IRF-3 and IRF-7 (Fitzgerald et al., 2003; Hemmi et al., 2004; Mcwhirter et al., 2004; Perry et al., 2004). Interaction between MAVS and TRAF3 is pivotal for the recruitment of both IKK complexes, while TRAF2 and TRAF6 are likely to be responsible for NF-κB activation (Xu et al., 2005; Khandekar et al., 2006). As a regulatory component, TANK and NAK-associated protein 1 (NAP1) are commonly involved in the TBK-1/IKK- iii-mediated activation of IRFs (Sasai et al., 2006; Guo and Cheng, 2007). IKK-γ (NEMO) serves as a regulatory subunit for the canonical IKK complex, which plays a role in the TBK-1/IKK- iii-mediated activation of IRFs (Zhao et al., 2007). However, FMDV 3C^pro^ can also target IKK-γ at the Gln383 residue and cleave off the C-terminal Zinc finger domain from IKK-γ, thereby disrupting the RIG-I/MDA5 pathway and contributing to the inhibition of IFN production (Wang et al., 2012). Most recently, crosstalk between the canonical IKK complex and non-canonical IKK complex exists in RLR-mediated signal transduction to a perform antiviral response (Fang et al., 2017). Collectively, these studies might imply that the immune systems have evolved a comprehensive network where various cytokines and signal molecules can perform cross-talk among different signal pathways to counteract the evasion of antiviral immune responses induced by viruses.

## Evasion of Antiviral Response of Type I IFN Mediated by FMDV

**FIGURE 2:**
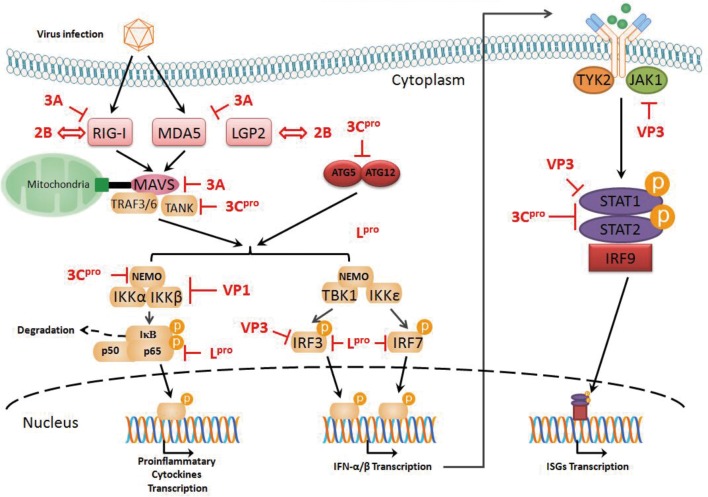
Evasion of antiviral response of type I IFN mediated by FMDV. FMDV Lpro can cleave the p65 subunit of NF-κB and inhibit IRF3/7, which promote the interferon-stimulated response element (ISRE). FMDV 3C^pro^ blocks the translocation of STAT1-STAT2 complex to the nucleus and suppresses the ISKE promoter. Recombinant VP1 can disrupt the formation of the canonical IKK complex to block NF-κB activity. VP3 can disrupt the assembly of the Jak1/STAT1 complex and inhibit IRF3 phosphorylation and dimerization. FMDV 2B can bind to RIG-I or LGP2 to impair related signal transduction and enhance viral replication. FMDV 3A protein can disrupt the transcriptional levels involved in RIG-I, MDA5, and MAVS and block the interaction between RIG-I/MDA5 and MAVS via its N-terminal region.

Upon viral infection, innate immune responses serving as the first line of defense against viruses can induce signal transduction involving IFN and finally the expression of type I IFN, which then contributes to an antiviral stage in the cells. However, many viruses have evolved strategies to evade the antiviral immune response and prevent IFN production. Picornavirus-induced host translation system shut-off has been known to result in IFNs and other cytokine suppression (Buenz and Howe, 2006). Due to these RNA viruses being armed with some viral proteases (i.e., L^pro^, 2A^pro^, and 3C^pro^), these proteases can target different signal transduction nodes mediated by IFN (Porter et al., 2006; Yang et al., 2007; Papon et al., 2009). FMDV is no exception and has evolved to use its production to evade the antiviral immune response against FMDV (**Figure 2**) and maximize viral replication and dissemination. In addition to promoting the generation of viral products with biological functions, FMDV L^pro^ and 3C^pro^ plays pivotal roles in disrupting the translational system of the host. For FMDV L^pro^, this protease is associated with translocation to the nucleus and cleavage of the p65 subunit of NF-κB (de Los Santos et al., 2007; Zhu et al., 2010). Although FMDV L^pro^ can inhibit IRF3/7 to promote the interferon-stimulated response element (ISRE) (Wang et al., 2011a,c), IRF9 can assist type I and III IFNs to perform antiviral response against FMDV infection (Stark et al., 2010; Perezmartin et al., 2012), implying that the host has evolved multiple immune pathways to meet with the evasion immune response induced by FMDV. Most recently, it has been reported that FMDV L^pro^ can bind to the host transcription factor ADNP (activity dependent neuroprotective protein) to suppress IFN and ISG transcription and enhance FMDV replication (Medina et al., 2017). FMDV 3C^pro^ specializes in cleaving FMDV polyprotein into viral proteins with biological functions and ruins eIF4G, eIF4A and histone H3 to block the translation system of the host (Falk et al., 1990; Li et al., 2001). In addition to inhibition of cell protein generation by 3C^pro^, it also disrupts the transcriptional levels of ISGs, blocks the translocation of STAT1-STAT2 complex to the nucleus and suppresses the ISKE promoter (Du et al., 2014). These biological functions of FMDV 3C^pro^ seem to ruin the IFN system against viral infection in the broad spectrum. Like the role of EMCV 3C^pro^ in impairing the interaction TANK and TRAF6-mediated NF-κB signaling (Huang et al., 2015), FMDV 3C^pro^ also cleaves TANK to block the non-canonical IKK complex signaling pathway, rather than FMDV L^pro^ only disrupting TANK binding activity. In general, protease encoded by FMDV can negatively regulate innate immune signaling by degradation of essential molecules in different pathway.

Apart from viral proteases of FMDV, other viral proteins play roles in viral immune evasion. VP1 and VP3 serve as viral structural proteins, which are the main components of the viral capsid (Mason et al., 2003). When the *vp1* gene was over-expressed in the human HEK293 cell line, VP1 and sorcin can reduce NF-κB at the transcriptional levels and weaken type I IFN activity; in addition, recombinant VP1 can disrupt the formation of the canonical IKK complex to block NF-κB activity (Li et al., 2013; Ho et al., 2014). When the *vp3* gene was over-expressed in the human HEK293T cell line, VP3 disrupted the assembly of the Jak1/STAT1 complex, suggesting FMDV VP3 impairs the type II IFN signaling pathway, inducing Jak1 degradation via a lysosomal pathway; Moreover, FMDV VP3 can inhibit IRF3 phosphorylation and dimerization, thereby contributing to the impairment of signal transduction involved in the type I IFN (Li et al., 2016a,b).

Turning to some non-structural proteins of FMDV, the 2B protein is capable of improving membrane permeability and carrying out cellular protein secretory pathway shut-off (Moffat et al., 2007). Recently, it has been reported that FMDV 2B can bind to RIG-I or LGP2 to impair related signal transduction and enhance viral replication, but the detailed mechanisms have not been established yet (Zhu et al., 2016, 2017). Enterovirus 71 2C proteins can bind to RelA (p65) and suppress IKKβ phosphorylation to disrupt the formation of the canonical IKK complex and impair NF-κB activation (Du et al., 2015; Li et al., 2016c). Since the 2C protein of picornaviruses are highly conserved (Gorbalenya et al., 1989), it is assumed that FMDV 2C should possess most of these activities involved in immune evasion. The FMDV 3A protein is a multifunctional non-structural protein in viral replication, virulence and host-specific genetic features (Mason et al., 2003; Ma X. et al., 2016; Bhatt et al., 2017; Bohórquez et al., 2017; Lotufo et al., 2017). For the role of FMDV 3A in the antiviral immune response, when 3A gene was over-expressed in the PK-15 cell line (swine) and the HEK293 cell line (human), 3A protein can disrupt the transcriptional levels involved in RIG-I, MDA5, and MAVS and block the interaction between RIG-I/MDA5 and MAVS via its N-terminal region (Li et al., 2016d). Although some studies involved in the structural and non-structural proteins of FMDV have noted that these viral proteins take part in immune evasion by disrupting various nodes along signaling transduction mediated by IFNs, the over-expressed individual protein with *in vitro* physical structure and biological function may not reflect the real situation in infected cells by FMDV.

## Conclusion

Based on recent knowledge on IFNs, it is now well established that these cytokines function in the innate immune response against viral infection. Collectively, even though a series of recent reports strongly indicate that the host has evolved a complex network related to signal transduction induced by IFN against FMDV infection and FMDV relies on its viral proteins to suppress or ruin antiviral immune responses induced by IFN systems, the detailed and precise regulatory mechanisms need to be elucidated. This field is thus at a stage where there is an urgent need for the better understanding of both the basic biology and therapeutic antiviral activity of IFNs, and for deep investigations of how those proteins, without protease activities, derived from FMDV infection can influence and control the IFN signaling transduction *in vivo*.

## Author Contributions

X-XM, L-NM, Q-YC, and L-JL wrote the draft manuscript. PM and Y-YW produced the figures. Z-RM and XC gave instruction and proof-read the article.

## Conflict of Interest Statement

The authors declare that the research was conducted in the absence of any commercial or financial relationships that could be construed as a potential conflict of interest.
